# Supplementary material for: The genome of opportunistic fungal pathogen Fusarium oxysporum carries a unique set of lineage-specific chromosomes
Source: Commun Biol. 2020 Jan 31;3:50. doi: 10.1038/s42003-020-0770-2 (PMC6994591; doi:10.1038/s42003-020-0770-2)
Supplement: Supplementary file 1 — Supplementary Information [file 42003_2020_770_MOESM1_ESM.pdf]

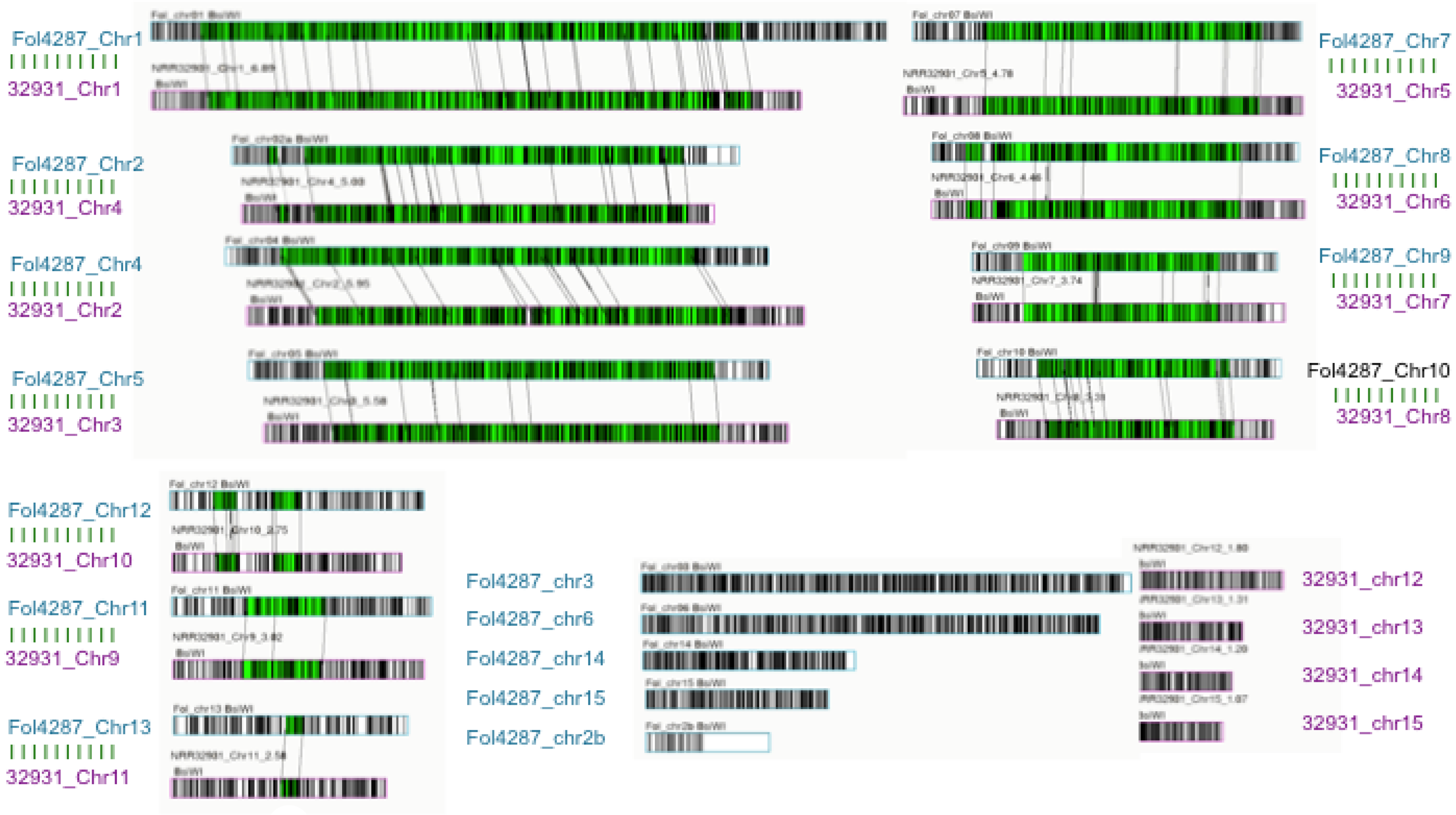

Supplementary Figure 1 Comparing optical maps of the human-infecting strain NRRL32931 (highlighted in purple) and the reference genome of Fol4287, a tomato wilt pathogen (highlighted in blue). Homologous fragments (highlighted in green) are detected among 11 homologous chromosomes that define the core genome, even though variations, mostly detected at chromosomal ends, exist even in the core genome. Distinctively, the human-infecting strain NRRL32931 contains four unique and smaller supernumerary chromosomes (chr12-15), and the Fol4287genome contains four SP-chromosomes and part of chr1 (linked) and chr2b (separated from the rest of chr2 after ribosomal repeats).

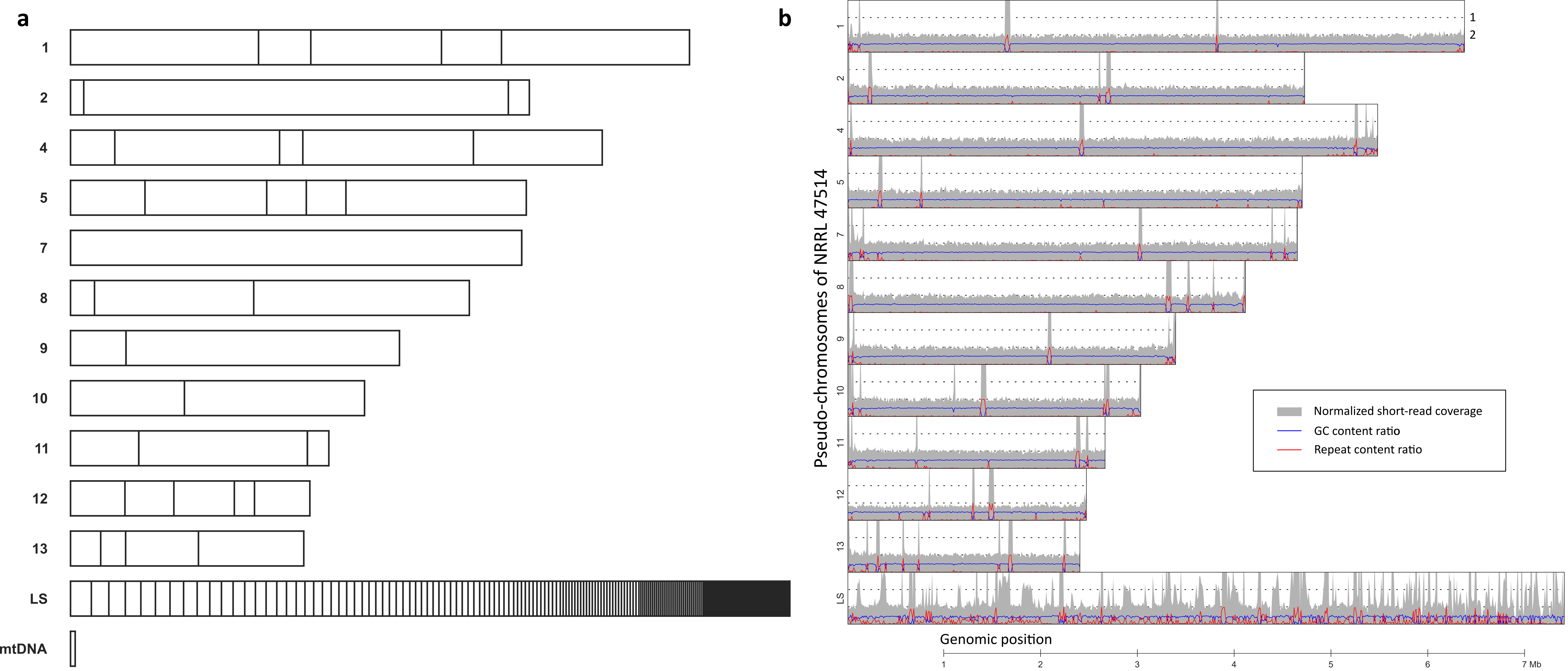

Supplementary Figure 2. *de novo* assembly of NRRL 47514 genome. (**a**) Assembled contigs of NRRL 47514 was compared to Fol4287 and put in pseudo chromosomes. (**b**) Illumina reads coverage distribution (grey area), GC content (blue lines), and Repeat content (red lines) of NRRL 47514 genome assembly. Median values for features over 10 kb windows are plotted in the contig order showed in a.

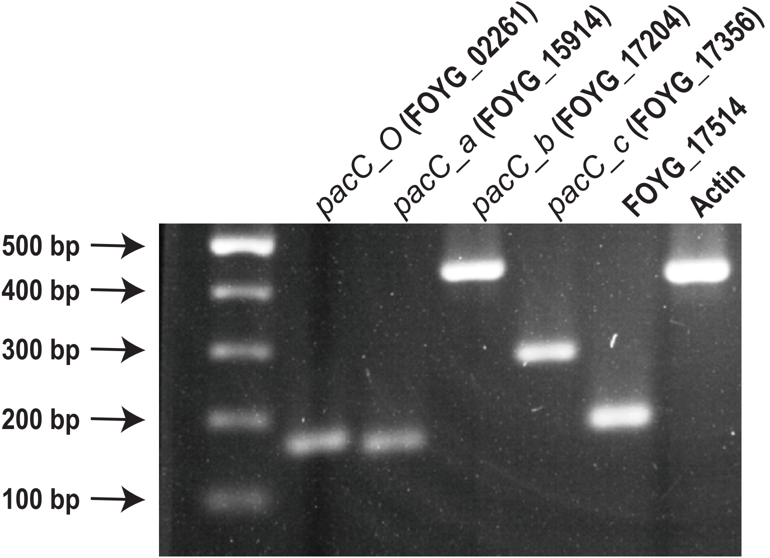

Supplementary Figure 3. PCR amplification of NRRL 32931 *pacC* genes.

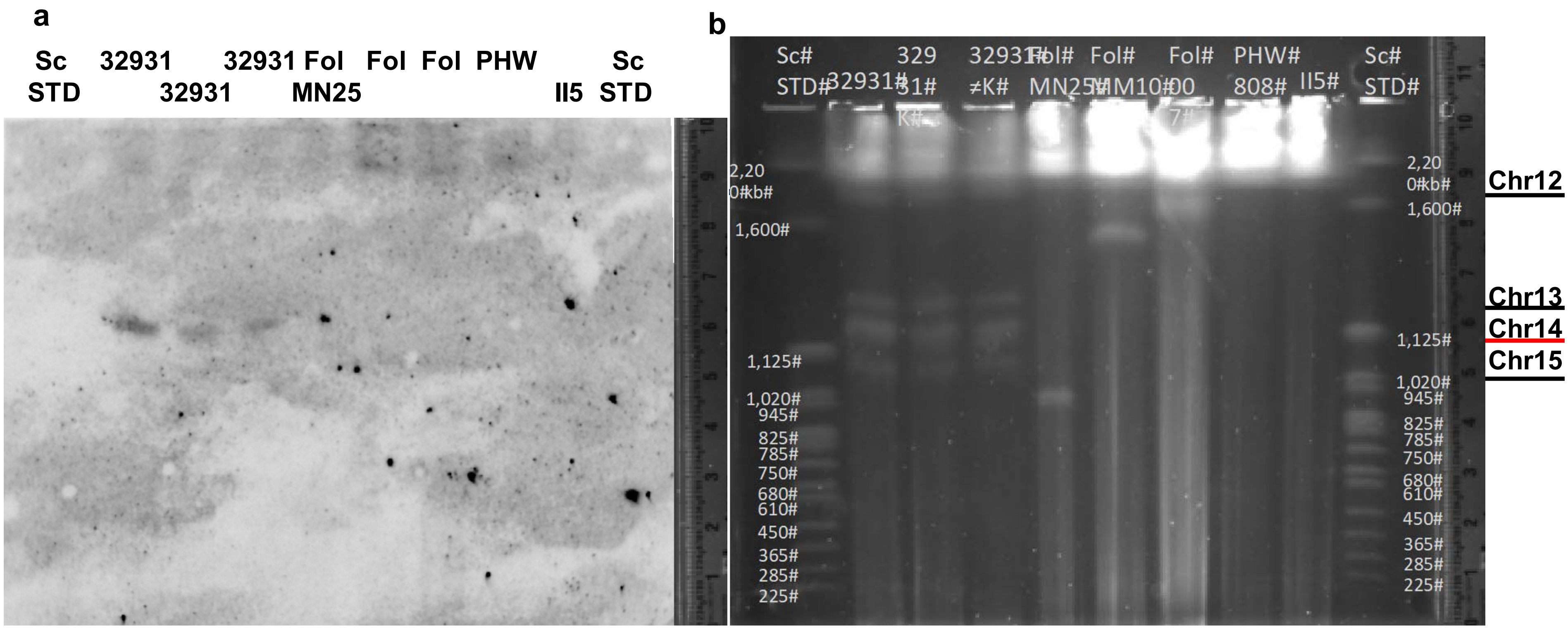

Supplementary Figure 4. a . Hybridization of *pacC_b* to Chr14 (1.2 Mb), one of the LS_chromosomes in NRRL 32931. **b.** Pulse field gel showing the chromosomal distribution of strain NRRL 32931 as shown in Figure 2. To separate chromosomes smaller than 2 Mb, protoplast plugs (4×10^8 ml^{-1} protoplasts) were run on a CHEF gel [1% Bio-Rad Pulsed Field Certified agarose (Bio-Rad, Philadelphia, PA, USA) in 0.5 × TBE] for 24 h using switch times between 60 and 120 s at 6 V cm^{-1} with an angle of 120°. Molecular size markers of *S. cerevisiae* chromosomes were used to assess chromosome size (Bio-Rad, Philadelphia, PA, USA). DNA was transferred to HyBond N+ (GE Health Care, Pittsburgh, PA, USA) using standard alkaline procedures. Probe labeling, hybridization and detection were performed as per the manufacturer's protocol for the AlkPhos Direct Kit with CDP-Star chemiluminescent detection reagent (GE Healthcare, Pittsburgh, PA, USA). The *pacC_b* probe was amplified by PCR using primers *pacC_b* F (TCGTACTCAAGGAGGCTTCCA) and *pacC_b* R (CTTAAAACGCCATTCTGGCT). Hybridization and washes were performed at 65°C.

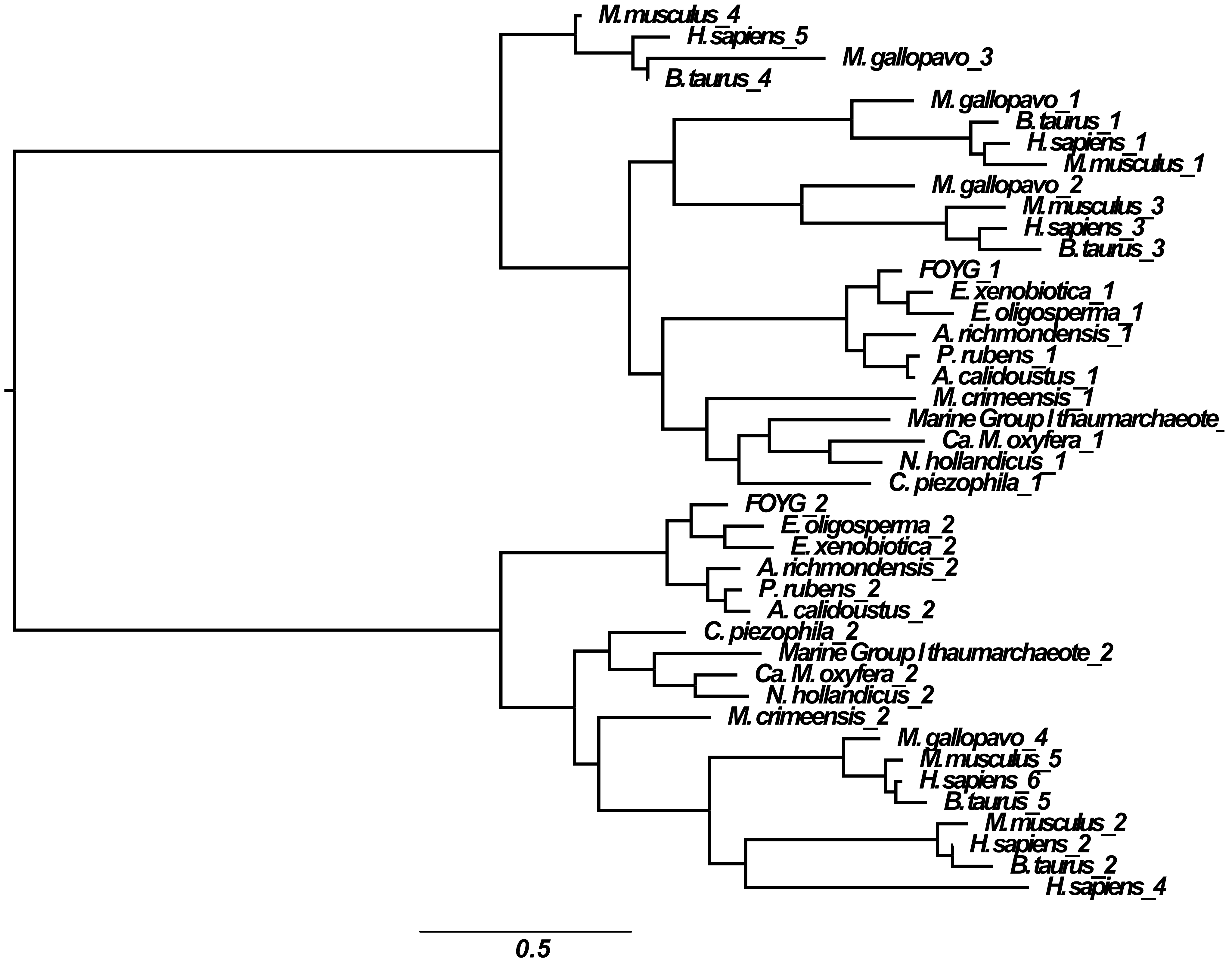

Supplementary Figure 5. Phylogenetic tree for multicopper oxidase domains in ceruloplasmins. All the domains can be divided into two groups. In addition, all the microorganisms contain just two domains from each group. However, for the animals, they have paralogous duplicates for two kinds of multicopper oxidase domains in each groups. This indicated that, for the ion binding function, the basic two conserved multicopper oxidase domains in ceruloplasmins are important.
