# Supplementary material for: The genome of opportunistic fungal pathogen Fusarium oxysporum carries a unique set of lineage-specific chromosomes
Source: Commun Biol. 2020 Jan 31;3:50. doi: 10.1038/s42003-020-0770-2 (PMC6994591; doi:10.1038/s42003-020-0770-2)
Supplement: Supplementary file 2 — Description of Additional Supplementary Files [file 42003_2020_770_MOESM2_ESM.docx]

**Description of Additional Supplementary Files**

**Supplementary Data 1.** Single-copy conserved genes used to infer phylogenetic relationships in Figure 1.

**Supplementary Data 2.** Alignment of 55 genes in FASTA file format.

**Supplementary Data 3.** Placement of supercontigs by comparing the *in silico* restriction map of the genome assembly with the restriction map used to create optical linkage groups.

**Supplementary Data 4.** Identification of NRRL 32931 supercontigs that belong to LS chromosomes using the “*Eliminating the Core Genome*” protocol.

**Supplementary Data 5.** Transcripts assembled based on RNA-Seq data, but absent in the genome annotation.

**Supplementary Data 6.** Rfam search results for the non-annotated, but transcribed sequences.

**Supplementary Data 7.** Transposon analysis by mapping the sequence reads to a set of annotated repeats and repeats identified using *de novo* repeat finding methods in the genomes of NRRL 32931 and NRRL 47514.

**Supplementary Data 8.** GO annotation of genes encoded in LS chromosomes.

**Supplementary Data 9.** List of potential horizontally transferred genes

**Supplementary Data 10.** Genes of the ergosterol biosynthesis pathway
