# Supplementary material for: The genome of opportunistic fungal pathogen Fusarium oxysporum carries a unique set of lineage-specific chromosomes
Source: Commun Biol. 2020 Jan 31;3:50. doi: 10.1038/s42003-020-0770-2 (PMC6994591; doi:10.1038/s42003-020-0770-2)
Supplement: Supplementary file 4 — Reporting Summary [file 42003_2020_770_MOESM4_ESM.pdf]

nature research

Corresponding author(s): Li-Jun Ma

Last updated by author(s): Dec 22, 2019

Reporting Summary

Nature Research wishes to improve the reproducibility of the work that we publish. This form provides structure for consistency and transparency in reporting. For further information on Nature Research policies, see Authors & Referees and the Editorial Policy Checklist.

Statistics

For all statistical analyses, confirm that the following items are present in the figure legend, table legend, main text, or Methods section.

n/a	Confirmed	
☒	☐	The exact sample size (*n*) for each experimental group/condition, given as a discrete number and unit of measurement
☐	☒	A statement on whether measurements were taken from distinct samples or whether the same sample was measured repeatedly
☐	☒	The statistical test(s) used AND whether they are one- or two-sided<br>*Only common tests should be described solely by name; describe more complex techniques in the Methods section.*
☐	☒	A description of all covariates tested
☐	☒	A description of any assumptions or corrections, such as tests of normality and adjustment for multiple comparisons
☐	☒	A full description of the statistical parameters including central tendency (e.g. means) or other basic estimates (e.g. regression coefficient) AND variation (e.g. standard deviation) or associated estimates of uncertainty (e.g. confidence intervals)
☐	☒	For null hypothesis testing, the test statistic (e.g. *F*, *t*, *r*) with confidence intervals, effect sizes, degrees of freedom and *P* value noted<br>*Give P values as exact values whenever suitable.*
☒	☐	For Bayesian analysis, information on the choice of priors and Markov chain Monte Carlo settings
☐	☒	For hierarchical and complex designs, identification of the appropriate level for tests and full reporting of outcomes
☐	☒	Estimates of effect sizes (e.g. Cohen's *d*, Pearson's *r*), indicating how they were calculated

Our web collection on statistics for biologists contains articles on many of the points above.

Software and code

Policy information about availability of computer code

Data collection	No software was used.
Data analysis	All commercial and open source softwares used in the manuscript are listed in Methods section.

For manuscripts utilizing custom algorithms or software that are central to the research but not yet described in published literature, software must be made available to editors/reviewers. We strongly encourage code deposition in a community repository (e.g. GitHub). See the Nature Research guidelines for submitting code & software for further information.

Data

Policy information about availability of data

All manuscripts must include a data availability statement. This statement should provide the following information, where applicable:

- Accession codes, unique identifiers, or web links for publicly available datasets
- A list of figures that have associated raw data
- A description of any restrictions on data availability

The raw sequencing data used for de novo whole-genome assembly of NRRL 32931 are available at NCBI (SRX101569, SRX101560, SRX101558, SRX081496, SRX081494, SRX081474). The accession numbers of NRRL 47514 (MRL 8996) whole-genome sequencing data are SRX6453258 (Illumina) and SRX6453257 (PacBio RS II). All Illumina HiSeq sequences of RNA-Seq are available with accession numbers SRX026545, SRX027736, SRX025824, SRX025823 at NCBI.

Field-specific reporting

Please select the one below that is the best fit for your research. If you are not sure, read the appropriate sections before making your selection.

☒ Life sciences ☐ Behavioural & social sciences ☐ Ecological, evolutionary & environmental sciences

For a reference copy of the document with all sections, see nature.com/documents/nr-reporting-summary-flat.pdf

Life sciences study design

All studies must disclose on these points even when the disclosure is negative.

Sample size	No statistical methods were used to determine sample size. Sample size was determined based on previous published researches in the field.
Data exclusions	No data were excluded.
Replication	Quantitative RT-PCR was performed with three biological replicates.
Randomization	Samples were selected randomly.
Blinding	Blinding was not relevant to this study.

Reporting for specific materials, systems and methods

We require information from authors about some types of materials, experimental systems and methods used in many studies. Here, indicate whether each material, system or method listed is relevant to your study. If you are not sure if a list item applies to your research, read the appropriate section before selecting a response.

Materials & experimental systems

n/a	Involved in the study	
☒	☐	Antibodies
☐	☒	Eukaryotic cell lines
☒	☐	Palaeontology
☒	☐	Animals and other organisms
☒	☐	Human research participants
☒	☐	Clinical data

Methods

n/a	Involved in the study	
☒	☐	ChIP-seq
☒	☐	Flow cytometry
☒	☐	MRI-based neuroimaging

Eukaryotic cell lines

Policy information about cell lines

Cell line source(s)	F. oxysporum NRRL 32931; F. oxysporum NRRL 47514;
Authentication	Cell lines were requested from ARS CULTURE COLLECTION (NRRL) and were frozen in dozens of tubes. We revived one stock each time and authenticated it by DNA sequencing.
Mycoplasma contamination	NA
Commonly misidentified lines (See ICLAC register)	No commonly misidentified cell lines were used in this study.
